# Recent Trends in the Use of Pectin from Agro-Waste Residues as a Natural-Based Biopolymer for Food Packaging Applications

**DOI:** 10.3390/ma13030673

**Published:** 2020-02-03

**Authors:** Cristina Mellinas, Marina Ramos, Alfonso Jiménez, María Carmen Garrigós

**Affiliations:** Department of Analytical Chemistry, Nutrition & Food Sciences, University of Alicante, ES-03690 Alicante, San Vicente del Raspeig, Spain; cristina.mellinas@ua.es (C.M.); marina.ramos@ua.es (M.R.); alfjimenez@ua.es (A.J.)

**Keywords:** pectin, food packaging, active compounds, agro-waste residues, circular economy

## Abstract

Regardless of the considerable progress in properties and versatility of synthetic polymers, their low biodegradability and lack of environmentally-friendly character remains a critical issue. Pectin is a natural-based polysaccharide contained in the cell walls of many plants allowing their growth and cell extension. This biopolymer can be extracted from plants and isolated as a bioplastic material with different applications, including food packaging. This review aims to present the latest research results regarding pectin, including the structure, different types, natural sources and potential use in several sectors, particularly in food packaging materials. Many researchers are currently working on a multitude of food and beverage industry applications related to pectin as well as combinations with other biopolymers to improve some key properties, such as antioxidant/antimicrobial performance and flexibility to obtain films. All these advances are covered in this review.

## 1. Introduction

Biopolymers are gaining their market share in the plastics industry by their intrinsic biodegradable character combined with interesting properties for specific applications. Biopolymers can be obtained/extracted from natural sources, biosynthesized by living organisms or chemically synthesized from biological materials [1]. In addition, their natural-based origin, i.e., from renewable sources represents a great advantage over plastic commodities since their use decrease dependence from petroleum while preserving and even improving important material properties. There has been an increasing interest for the use of biopolymers in packaging, medicine, agriculture, and other sectors. Different types of carbohydrates, such as starch and cellulose, as well as other polysaccharides, such as alginates and pectin, as well as their combinations with animal-protein-based biopolymers, such as silk, wood, gelatin, collagen, chitosan/chitin, gums, plant-based proteins and lipids offer the possibility of rendering interesting applications for these advanced sectors. All these biopolymers offer interesting advantages in their use, such as their renewable origin, biocompatibility, barrier properties to moisture and/or gases, non-toxicity, non-polluting characteristics, mechanical integrity and relative low cost.

The increase in the use of biopolymers has caused that their global market is expected to reach around 10 billion US Dollars by 2021, growing by almost 17% over the forecast period 2017–2021. Western Europe covers the largest market segment, accounting for 41.5% of the global market while other regions are rapidly increasing their market share [2].

**Figure 1:**
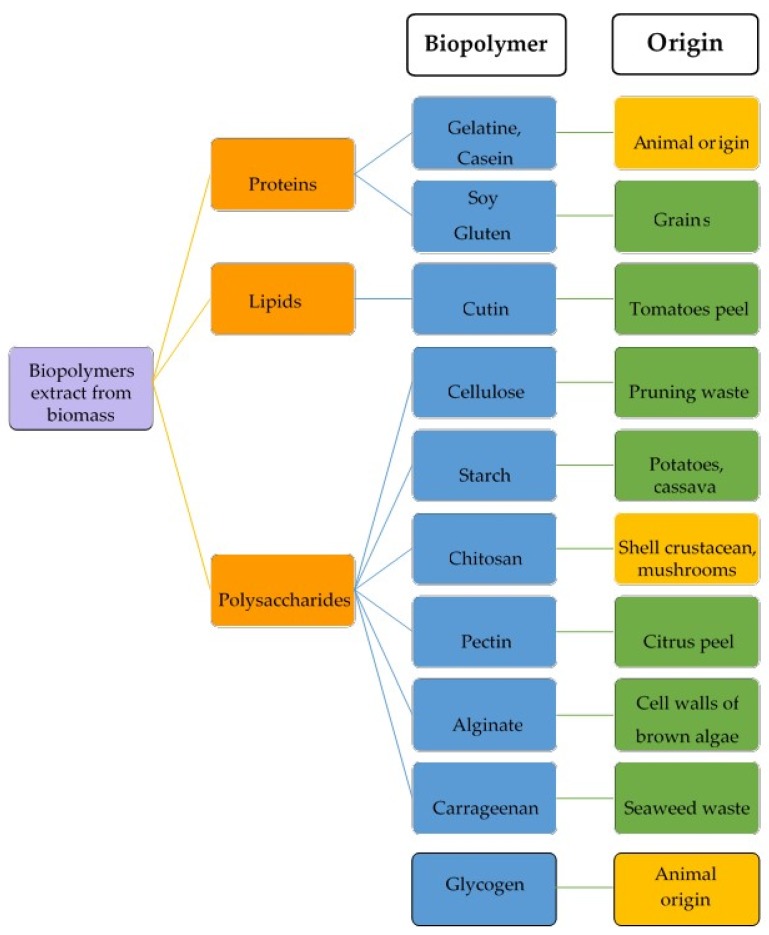
Different types of biopolymers obtained from animal and vegetable wastes.

In addition, another important possibility offered by the use of biopolymers is their potential to be synthesized from the non-edible parts of plants or animals, avoiding the risk of depleting food from local communities, most of them in under-developed regions. Figure 1 shows the main types of biopolymers that can be obtained from biomass waste as well as some examples of their sources. They can be divided into three large groups: proteins, lipids and polysaccharides. The protein-based biopolymers can be obtained from both animal and vegetable wastes. For example, slaughterhouse wastes are a good source of proteins from animal origin, like gelatin. These wastes comprise the inedible tissues/parts of the animals slaughtered for the production of meat [3]. Among the proteins from plant origin, soy protein isolate is a good option to develop new materials due to its composition and excellent processing ability by gelling, emulsifying ability and water and oil holding capacity [4].

Lipid-based polymers have been used in the last few years in food packaging or 3D printing materials. Their extraction from the natural sources is a necessary step to render isolated fatty acids to be further used in esterification reactions. For example, cutin extracted from tomato by-products [5] and lipids extracted from algae [6] have shown great potential to obtain specific fractions for the production of films with high barrier to water due to the repulsion caused by their high hydrophobic behaviour.

Polysaccharide-based polymers are the last group in Figure 1. These biopolymers are characterized by their biodegradable, biocompostable, sustainable and non-toxic characteristics. Additionally, polysaccharides are more thermally stable than other biopolymers, like lipids and proteins, since they are not irreversibly denatured via heating. However, their main disadvantages are the high sensitivity to moisture and low mechanical resistance [7]. In order to limit these problems, two different approaches have been proposed: the incorporation of different reinforcing additives to the polysaccharides matrices [8,9] and the combination with different polymers to obtain blends [10,11] or multilayer films [12,13]. The improvement in polysaccharides properties have permitted the extension of their use by the food industry in the last few years [14,15].

Pectin is one of the major structural polysaccharides present in many higher plant cells allowing primary cell wall extension and plant growth. It could be extracted and applied as an anionic biopolymer, soluble in water. A large number of recent articles have highlighted the advantages of using pectin over conventional polymers. Therefore, pectin is increasingly important for a multitude of food packaging applications, such as a thickening and gelling agent, colloidal stabilizer, texturizer, and emulsifier [16,17,18], a coating on fresh and cut fruits or vegetables [19,20,21] and as micro and nano-encapsulating agent for the controlled release of active principles with different functionalities [22]. Rodsamran et al. [16] reported that bioactive pectin films can retard soybean oil oxidation during 30 days of storage. Furthermore, Sucheta et al. [19] found that a pectin-corn flour-based coating significantly reduced the weight loss and decay per cent of tomatoes, delaying respiration with retention of biochemical quality of tomatoes. Additionally, polymeric blends of hydrocolloids obtained from chia seeds and apple pectin where developed with the aim to obtain antioxidant polymer blend films using the 2,2-diphenyl-1-picrylhydrazyl (DPPH) assay to estimate their antioxidant activity [10].

Pectin has been also extracted from waste biomass by using innovative methods, contributing to waste management in agriculture and food processing industries. Different pectin sources can be used, such as by-products of juice manufacturing as well as orange, mango, banana, lime and pomegranate peels and seeds. Therefore, this review aims to present and discuss the potential of pectin as a bio-based material in food packaging applications by its efficient extraction from waste biomass, while addressing a solution to the important environmental problems caused by the disposal of residues and by-products in the food sector.

## 2. Pectin

### 2.1. Pectin Structure

**Figure 2:**
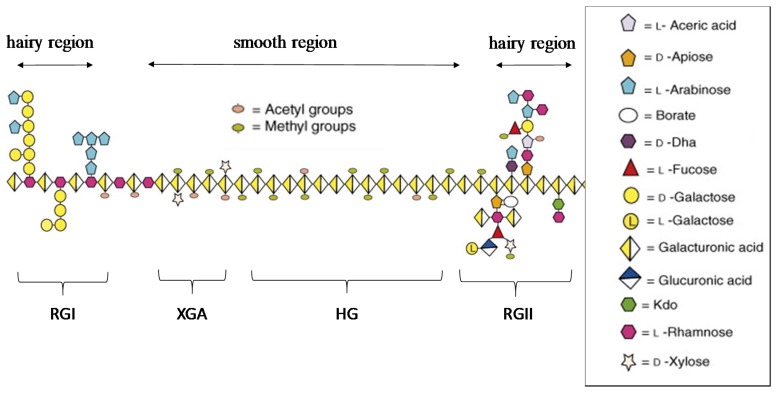
Schematic pectin structure, adapted from [23].

Pectin is a complex heteropolysaccharide and a major multifunctional component of the cell wall in many terrestrial plants. It is usually found in association with other compounds like cellulose, lignin or polyphenols present in the cell wall of plants [22]. Pectin is mainly composed of galacturonic acid units (Figure 2). The carboxyl groups of uronic acid residues can be present in different forms in the polymer structure, either free or as a salt form with sodium, calcium or other small counter-ions. In some cases, they can be also present as naturally-esterified groups, particularly with methanol, depending on the pectin source and/or the extraction method. Due to the presence of free carboxyl groups, pectin solutions exhibit acidic pH values. Galacturonic acid comprises approximately 70% of the pectin composition, depending on the plant species, and all the pectic polysaccharides contain galacturonic acid linked at the O-1 and the O-4 positions [23]. Pectin has a linear anionic backbone which regions showing no side chains known as “smooth regions” and regions with non-ionic side chains known as “hairy regions” [24].

Different pectin structural domains may be distinguished (Figure 2), influencing their properties depending on pectin proportions [23].

Homogalacturonan (HG): HG is the major domain of pectin in cell walls of plants, representing approximately 65% of the total pectin content. It is formed by galacturonic acid residues, linked by α (1→4) bonds, and their carboxyl groups are partially methyl esterified at position 6. Additionally, this domain may be acetylated at position 2 or 3 depending on the origin of the pectin. These domains are the main constituents of the above-mentioned “smooth regions” [24].

Rhamnogalacturonan I (RG-I) contains about 20%–35% of the total pectin and shows a more complex structure than HG. It contains repeated units of disaccharides consisting of L-rhamnose and galacturonic acid that can be also acetylated in the positions 2 or 3. It could have up to 100 units of (1,2)-α-L-Rha-(1,4)-α-D-GalA. In addition, large amount of L-rhamnose structures are substituted at O-4 by different neutral sugars such as L-galactose and L-arabinose [23].

Rhamnogalacturonan II (RG-II) contains about 10% of the total pectin and it is the structurally more complex component. Despite it is a relatively minor component in the pectin chain, RG-II plays a central role in the structure of plant cell walls. Small structure modifications of RG-II lead to reductions in the dimers formation and they can cause severe growth defects. So, dimerization of RG-II in the cell wall may be crucial for the normal growth and development of plants. This domain is composed of an HG backbone of (at least eight) 1,4-linked α-d-GalA residues decorated with side branches consisting of different types of sugars (rhamnose, fucose, xylose, galactose, apiose or aceric acid) in over 20 different types of linkages [23,25,26].

It is generally believed that the pectic polysaccharides are covalently bonded with high crosslinking densities since harsh chemical treatments or digestion by pectin-degrading enzymes are required to isolate HG, RG-I, and RG-II from each other. In addition, it has been reported that other components, such as xylogacturonan (XGA) and apiogalacturonan (AP), could replace the galacturonic acid units in some parts of the pectin chain [23]. The complexity of the pectin structures increases since it can be changed during the plant storage, extraction and processing, resulting in modifications of pectin functionalities and hindering its structural elucidation. It has been reported that the variations in chain lengths in each of the different domains are not the same, because HG and RGII have a highly homogeneous structure while RGI exhibits a wide heterogeneity in its composition [26].

### 2.2. Type of Pectins

**Figure 3:**
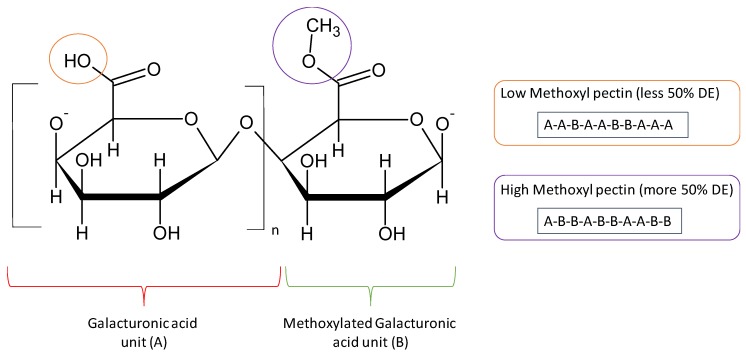
Structure of low and high methoxyl pectins.

The degree of esterification (DE) is an important parameter for the definition of the pectin applications and it is defined as the percentage of carboxyl groups esterified present in the structure of pectin. DE is often used to classify the different types of pectins (Figure 3). Depending on the DE different emulsifying, texturizing and gelling properties are observed. In general, when the DE increases, the water solubility decreases due to the hydrophobic nature of esters with long hydrocarbon chains. In contrast, when the DE increases the gelation rate also improves resulting in rapid gelation pectins [27]. Furthermore, the amount and composition of neutral sugars and the overall molecular weight of pectins have a great influence in their rheological properties [28].

High methoxyl pectin (HMP) shows DE higher than 50% and it is mainly used in the food industry by its thickening and gelling properties. It has been reported that HMP requires high amount of sugars for gelation and it is very sensitive to acidity [29]. HMP forms gels at low pH values and high concentrations of soluble solids due to the presence of hydrogen bonding and hydrophobic interactions between the pectin chains. Neutral sugars like sucrose play different roles in gelation through regulation of the hydrophobic interaction or directly binding to the polymer chains of HMP. Gels are formed when HG portions are cross-linked to form three dimensional crystalline networks in which water and other solutes are trapped [30]. The mechanism of formation of HMP gels is complex and it has been the subject of many investigations in the last decades. The glass transition theory has been proposed to explain the formation of HMP gels. Due to the high viscosity of molecules an arrest of the system kinetics occurs, giving as a result the formation of the gel due to the increased concentration of co-solutes and decrease in the water content. The transition from sol to gel behaviour is due to the combined effect of HMP and sucrose at pH 3, and occurs when excluded volume effects and attractive interactions are capable to give rise to an incipient three-dimensional network [31]. In addition, the effect of monovalent cations has been evaluated under different alkaline conditions (NaOH and KOH) at different pectin concentrations. It was suggested that HMP gel is formed through different mechanisms, such as de-esterification, self-aggregation, and entanglement under alkaline conditions. Na^+^ or K^+^ bind to dissociated carboxyl groups in HMP due to electronic attraction and this behaviour enables HMP molecules to move closer to each other, thereby improving gel network formation [32].

The emulsifying properties of HMP have been investigated by Jiang et al. in binary water-ethanol systems. They suggested that ethanol reduces the electrostatic repulsion and promotes pectin aggregation [33]. When HMP was mixed with the water-ethanol mixtures, the helix structure was broken, and electrostatic repulsion decreased. The compact and hydrophobic conformation enables pectin to adsorb better on the oil-water interface. The obtained emulsion showed good stability when using 21% of ethanol in the mixture.

Low methoxyl pectin (LMP) shows DE lower than 50% and it is generally formed by the de-esterification of HMP. Different agents can be used for the preparation of LMP from HMP, such as alkalis like sodium hydroxide or ammonia, enzymes (pectin methyl esterase) and concentrated acids [34].

LMP is widely used by the food industry to form low sugar-content jams as it does not require large amounts of sugar for gelation. It shows less sensitivity towards acidity and requires Ca^2+^ ions to form gels. The gelation mechanism in LMP is mediated by the formation of calcium bonds between two carboxyl groups from two chains in close contact [28]. Recently, Han et al. studied the effect of the calcium concentration, pH, soluble solids, and pectin concentration on the gel strength of LMP gels and they proposed different mechanisms of formation of pectin gels based on their rheological properties [35]. They observed that pH values close to the isoelectric point (pH = 3.50) and high calcium concentrations enhanced the storage modulus and gel strength by formation of calcium bridges at dissociated carboxyl groups. In addition, the sucrose content improved the gel strength because the neutral sugars provide hydroxyl groups to stabilize the gel and contribute to the formation of hydrogen bonds to immobilize free water. On the other hand, the formation of LMP gels under alkaline conditions was tested by Yang et al. [36]. They suggested that LMP can form relatively stable gels in the pH range of 3.5–9.5 using NaOH as pH regulator. In addition, they evaluated the effect of calcium concentration in the thermal and structural properties of the LMP gels and they concluded that the presence of calcium ions not only reduced the thermal stability but also the crystalline degree of LMP.

## 3. Sources of Pectin

Due to the high potential of pectin-based polymers, the extraction of pectin from biomass waste has been widely studied. Table 1 summarizes several published works based on the extraction of pectin from agro-waste sources.

The peels of citrus fruits have been reported as the main source to obtain pectin at the industrial scale due to their good properties and high extraction yield. Hydrothermal extraction is the most usual method to obtain pectin from orange peels and it involves high temperatures (75–95 °C) and extraction times (60–300 min). Additionally, in all cases, the hydrothermal extraction of pectin takes place under acidic conditions using water as solvent. Pectin is very soluble in water and the acid medium decrease the presence of other compounds like polyphenols increasing extraction yields and helping to maintain the quality of the extracted pectin [38,39,40,41]. Other methods have been tested to reduce extraction times in citrus by-products. For example, microwave-assisted extraction (MAE) has been used in lime [64] and pomelo peels [65] reducing the extraction times to five and two minutes, respectively. However, high microwave powers (700–1100 W) were required to achieve these results. The hydrodynamic cavitation method was also used to obtain pectin derived from orange peel waste. Although a large decrease in the amount of solvent (2.86 mL/g of dry waste) was observed, long extraction times were also needed (270 min) [55].

The use of other sources to obtain pectin-based polymers in good grade and quality has been proposed in the last few years, such as eggplant peel [37], chamomile waste [45], cocoa pod husk [59,66], banana peel [49], mango peel [50,61,67] or tomato husk [28]. Tropical fruits have been also studied in the last years to obtain HMP. For example, passion fruit rind [54,62], durian rind [46] or jackfruit peels [51,52,63] have been proposed as interesting sources of pectins. Hydrothermal extraction is also the most used method in these types of wastes. Ultrasound-assisted extraction (UAE) has been also tested in passion fruit rind using 450 W and a water to dry sample ratio of 20 mL/g for 10 minutes. Results showed that the obtained pectin was mainly formed by homogalacturonans. Furthermore, their high degree of methylation indicated that the passion fruit pectin could be applied in gel forming products [62].

The use of innovative and sustainable extraction techniques is heading towards the study of hybrid techniques with the objective of combining their advantages, such as in the case of MAE and UAE. Pectin has been obtained from sisal waste by the combination of enzymatic and ultrasonic processes as an efficient strategy for the production of high-quality pectins since the enzymatic treatment disrupt the links between cellulose and xyloglucans in the cell wall of sisal and then the ultrasonic treatment produces mechanical destruction of the sisal structure to improve the release of pectin [57].

Finally, the introduction of new extraction techniques can be a great initial investment for companies since they offer the possibilities to get specific extractions of high added value purified compounds, although the costs of microwave or ultrasonic based equipment are higher than those of conventional extraction equipment, but in the long term, these devices are more profitable since the energy consumption, extraction time and the amount of expensive reagents used during pectin extraction are reduced [68,69].

## 4. Pectin-Based Materials for Food Packaging Applications

Pectin is a versatile compound that can be used to develop different materials in many food applications such as thickening and gelling agent, colloidal stabilizer, texturizer and emulsifier. These important applications are not limited to food processing, but also to packaging, coatings on fresh and cut fruits or vegetables and as microencapsulating agents (Table 2). Pectin is soluble in pure water and insoluble in organic solvents. Moreover, when dry pectin is mixed with water it tends to hydrate very rapidly, forming clumps. This behaviour is due to the formation of dry spheres of pectin contained in a highly hydrated outer coating. In order to eliminate these clumps, a vigorous and long agitation time is required [70]. In general terms, diluted pectin solutions present a Newtonian behaviour, but at high concentrations they show non-Newtonian behaviour, corresponding to pseudo-plastic characteristics. It was observed that the decrease in solubility and increase in viscosity contribute to increase the gelation capacity, i.e., the pectin concentration has a positive effect in gelation capacity and viscosity but a negative effect in solubility. Although it was stated that pectin properties are mainly dependent on structure, particularly DE [22,71], film forming, gelling and emulsifying properties should be also considered.

### 4.1. Pectin-Based Films

Casting is the most used technique to obtain pectin-based films [10,73,74,75,79]. Pectin solutions (around 2–3 wt%) are mixed with the appropriate amount of plasticizer, commonly glycerol [95]. Then, the film forming solution is dried under controlled conditions of temperature and humidity forming a thin film. The incorporation of active agents, such as antimicrobial and/or antioxidant compounds, is performed after the incorporation of the plasticizer to obtain good compatibility between all components during the film processing [96,97,98]. Recently, Gouveira et al. [99] have reported the successful production of pectin-based films by using thermo-compression moulding of raw pectin with a natural deep eutectic solvent. The visual aspect of the obtained films was acceptable, since they were yellowish, visually homogenous, semi-transparent and without apparent pores, also showing high tensile strength and water resistance.

Pectin offers good compatibility with other biopolymers, such as proteins [73], lipids [100], other natural polysaccharides [101] or even synthetic biopolymers [82]. All these combinations represent alternatives when considering the final application of the obtained films. Both types of pectin (HMP and LMP) can form thin films under specific conditions. For example, LMP has been used as an appropriate matrix in new antioxidant systems with ascorbic acid as active additive by using casting as the processing method [72]. LMP was heated to 90 °C and then, glycerol and ascorbic acid were incorporated to the solution. Finally calcium chloride was added as the crosslinking agent to permit the formation of consistent and homogeneous pectin-based active films. In contrast, the addition of calcium ions is not necessary to develop films based on HMP, but low pH values and high sugar concentrations are needed to produce thin films. Nisar et al. [74] produced HMP films with antimicrobial properties incorporating clove essential oil by the casting method. Film forming solutions (3% *w*/*v*) were prepared by rehydrating pectin in sterile deionized water for 12 h at 20 °C. Glycerol was used as plasticizer at 30 wt % with magnetic stirring at 70 °C while pH was adjusted to 4.5. The clove essential oil with an emulsifier to improve the oil dispersion in the film aqueous solution were incorporated into the film forming solutions at different concentrations. A great integration of the clove essential oil into the polymer matrix was observed with a positive significant influence on the physico-chemical and functional properties, in particular barrier, mechanical, antioxidant and antimicrobial.

Marjoram, mint and rosemary essential oils are some of the active additives incorporated into the pectin matrix to get functionalities to these biopolymer materials. Almasi et al. [75] evaluated the effect on physico-chemical properties of marjoram essential oil in pectin films for food packaging applications. In order to prevent the degradation of the highly volatile essential oil and to control its release into food, it was incorporated using nanoemulsions and Pickering emulsions. Both types of emulsions combined the use of the essential oil and a low molecular weight surfactant with whey protein isolate or inulin as nanocarriers. Results obtained through X-ray diffraction (XRD), Fourier transformed infrared spectroscopy (FTIR) and field emission scanning electronic microscopy (FESEM) confirmed the high compatibility between pectin and both emulsions. The encapsulation of the essential oil through Pickering emulsions provided significantly slower releasing rates through the films when compared to nanoemulsions. For these reasons, authors concluded that the active pectin films containing Pickering emulsions showed the best potential to be used in active food packaging due to the slow release of the essential oil increasing food shelf-life. On the other hand, the synergic effect between mint and rosemary essential oils and nisin was investigated by Akhter et al. [78] using chitosan, starch and pectin blends. These authors concluded that the inclusion of rosemary essential oil and nisin improved the water barrier properties, tensile strength and thermal stability of the active biocomposites. Additionally, the combination of these compounds in a pectin matrix showed high antimicrobial action against some pathogenic strains (*Bacillus subtilis*, *Escherichia coli* and *Listeria monocytogenes*).

Furthermore, extracts derived from plants have been proposed to improve the functional properties of pectin. For example, tea extracts were incorporated into a HMP/Glucomannan blend. The influence of the addition of tea extracts at different concentrations (from 1% to 5% wt % on a dry basis) on the structural and physical properties of the blend, as well as on the antioxidant and antimicrobial activities were evaluated [76]. The authors found that concentrations of tea extract lower than 2 wt % improved all these properties but the effect was negative at high concentrations since some aggregation in the biopolymer macromolecules was observed. Red cabbage extract has been proposed for the development of a smart film based on HMP for meat and fish products. [80]. Red cabbage extract is rich in anthocyanins, showing the ability to change the colour of the biopolymer matrix at different pH values. It is known that the degradation of animal proteins produces an increase in pH due to the liberation of nitrogen compounds that can be monitored using a colorimetric sensor based on pectin and the red cabbage extract, offering innovative applications of pectin to the food industry [80]. These results showed the significant colour change in edible films when they are exposed to the headspace of meat and fish products at 21 °C and 4 °C, respectively.

The physical and functional properties of pectin-based films can be also modified by the combination of commercial pectin with corn flour and beetroot powder to minimize post-harvest decay, reducing ripening and improving sensorial properties of tomatoes [19]. In this study, results showed that pectin-based films protect from losses of polyphenols improving the antioxidant activity of these materials. In addition, other properties are modified due the presence of the edible coating. Pectin can modify the atmosphere around the fruit and/or vegetables, altering oxygen levels inside the fruit, retarding production of ethylene and, thus, limiting their physiological decay. In this work, pectin-based films showed low hydrophobicity to get optimum gas and water vapour permeability; reducing the ripening induced quality degradation in terms of texture and loss of bioactive compounds during storage.

Finally, the incorporation of nanoparticles to improve the physical and functional properties of pectin-based films has been evaluated. Biocomposites formed by a biopolymer matrix with metal or metal oxide nanoparticles are gaining importance in active food packaging since they could play a double role. On one hand, nanoparticles can act as nanofillers to enhance the mechanical and barrier properties of the biopolymer matrix and, on the other hand, they can interact directly with food due to their potential antimicrobial/antioxidant activity [102]. The effect of silver nanoparticles (AgNPs) has been tested in pectin, pullulan (a polysaccharide produced by fermentation by the *Aureobasidium pullulans* fungus), and pectin/pullulan blends [77]. Silver nanoparticles improved the mechanical properties of pullulan/AgNPs and pullulan/AgNPs/pectin composites while also showing high antimicrobial activity against foodborne pathogens, especially *Salmonella Typhimurium*, *Escherichia coli* and *Listeria monocytogenes*. AgNPs have been also proposed to develop nanocomposites based on pectin to be used as coatings for other polymer matrices with the aim to improve their barrier and mechanical properties as well as providing antimicrobial/antioxidant properties. Nanocomposites based on pectin with AgNPs and laponite have been evaluated to get a significant reduction in the oxygen transmission rate and water vapour transmission rate respect to neat polypropylene films taken as control [82]. The application of these new films showed antimicrobial activity against Gram-negative and Gram-positive bacteria, *Escherichia coli* and *Staphylococcus aureus*, respectively.

Other types of nanoparticles have been tested in pectin as the polymer matrix. Titanium oxide nanoparticles (TiO_2_NPs) were incorporated at low concentrations (0–2 wt %) into biodegradable starch–pectin (3:1) films to improve their mechanical and barrier properties as well as their potential as antioxidant systems for food packaging applications [103]. In addition, visible and UV radiation was completely absorbed or scattered in these films by the addition of TiO_2_NPs to get starch-pectin films with potential as UV screening packaging materials. On the other hand, the addition of halloysite nanotubes (HNT) offers great advantages to develop advanced food packaging materials. The effect of HNTs with salicylic acid [104], rosemary [97] and peppermint [105] essential oils in pectin films has been reported. HNTs showed high compatibility with pectin films improving their mechanical, thermal and moisture barrier properties [97,104]. The antimicrobial performance of these films was also improved due to the increase in the release rate of active compounds [97]. In fact, the antimicrobial activity of pectin-based films against Gram-negative *Escherichia coli* ATCC 25922, *Salmonella Typhimurium* ATCC 14028, *Pseudomonas aeruginosa* ATCC 10145, and Gram-positive *Staphylococcus aureus* ATCC 29213 was studied by the disk diffusion method, suggesting the effective antimicrobial properties of these functionalized films [104].

### 4.2. Emulsions and Gels

Pectins are widely used in the food industry as emulsifier and gelling agents. The ability of pectins to form gels under specific conditions has been used to obtain aerogels [86,106], hydrogels [87,107] or oleogels [88,108]. In particular, hydrogels are the most popular gel compositions used in food packaging, since they are able to absorb large amounts of water or other biological fluids inside their structure. For example, Torpol et al. studied the encapsulation of two different antimicrobial compounds: garlic and holy basil essential oils in chitosan-pectin hydrogel beads [87]. The entrapment of essential oils in the matrix structure was successful and it showed antimicrobial capacity against *Bacillus cereus*, *Clostridium perfringens*, *Escherichia coli*, *Pseudomonas fluorescens*, *Listeria monocytogenes* and *Staphylococcus aureus*, but not against *Lactobacillus plantarum* and *Salmonella Typhimurium.* Hydrogel coatings have been also proposed to reduce the deterioration of fresh fruit, meat or fish, as they can provide a semi-permeable protection to gases and water vapour and some other environmental factors that could damage food. By promoting food perspiration, these films also help to reduce enzymatic browning and water loss. Furthermore, this protection may also be enhanced by the addition of other ingredients, such as minerals, antioxidants, nutrients, vitamins or probiotics [109]. On the other hand, when the extraction of solvents at their supercritical state is produced in hydrogels or alcogels, the resultant material is called aerogels. Due to their unique properties, such as high porosity, high specific surface area, low relative density and thermal conductivity, these pectin-based biopolymers represent an innovative approach as advanced materials for food packaging since they can be used as internal layers, oxygen scavengers or drug delivery systems [110,111]. Recently, pectin-based aerogels have been developed for the storage of temperature-sensitive food. In this regard, TiO_2_ nanoparticles were incorporated into the pectin matrix to improve the mechanical, thermal and antimicrobial properties of pectin when compared to control films [86].

As it has been mentioned above, pectin can be used as nanoemulsions, which are kinetically stable, but thermodynamically unstable, systems whose production requires emulsifiers to stabilize the dispersed phase [93]. Different types of essential oils have been encapsulated using nanoemulsions to delay and control the release processes. Several authors have studied the use of nanoemulsions in pectin matrices with different essential oils extracted from curcumin [91], lemongrass [92,93] and oregano [92]. Although essential oils are especially interesting in food packaging applications due to their antioxidant and/or antimicrobial properties, Mendes et al. [93] incorporated pectin nanoemulsions with the lemongrass essential oil into cassava starch film to improve the biodegradation rate of these formulations. Results obtained for the film with nanoemulsions showed a suitable degradation in vegetal compost, ensuring their complete biodegradation in a short time increasing their potential application in the food industry.

## 5. Conclusions

Researchers and scientists have achieved great success in the development of new systems based on pectins, as a natural bio-based biopolymer that can be obtained from agro-waste products, contributing to the implementation of the circular economy concept by improving waste management. This review article has considered the latest results obtained by researchers on the extraction, functionalization and potential applications in the food industry (including packaging), such as the production of films, emulsions and gels. However, due to the variability in the pectin structure the final application of pectin matrices is very diverse but very promising in many fields related to food packaging, particularly when active formulations are searched. Further studies on pectin matrices and optimization of polymer processes will be needed to better control the resulting pectin-based products.

## Figures and Tables

**Table 1:** Different raw materials and extraction methods to obtain natural pectin.

Raw Material	Extraction Method	Conditions				Ref.
		T (°C)	Time (min)	LSR (mL/g)	Other Variables	
Eggplant peel waste	HAE	90	30	40	-	[37]
Orange peel waste	HAE	80	60	17.1	pH: 1.5	[38]
Orange peel waste	HAE	75	300	20	pH: 2.5	[39]
Orange peel waste	HAE	95	120	6	pH: 1.6	[40]
Pomelo peel waste	HAE	90	120	30	pH: 2	[41]
Pomegranate peel waste	HAE	70	120	10	-	[42]
Apple peel waste	HAE	85	120	25	-	[43]
Cashew apple pulp	HAE	100	120	5.15	-	[44]
Chamomile Waste	HAE	90	60	20	pH: 1.2	[45]
Durian rind waste	HAE	85	60	9	pH: 2.5	[46]
Hibiscus (*sabdariffa* L.)	HAE	100	30	20	pH: 2.5	[47]
Banana peel waste	HAE	86	360	50	pH: 2	[48]
Banana peel waste	HAE	90	30	20	pH: 1.5, 6	[49]
Mango peel waste	HAE	90	120	20	-	[50]
Jackfruit peel waste	HAE	138	9	17	-	[51]
Jackfruit peel waste	HAE	90	60	10	-	[52]
Jackfruit peel waste	HAE	90	60	20	-	[53]
Passion fruit rind	HAE	98	90	50	-	[54]
Tomato husk waste	HAE	100	15–25	30	-	[28]
Orange peel waste	HC	14.6–96	270	2.86	-	[55]
Artichoke (*Cynara scolymus* L.)	EAE	50	2880	15.4	pH: 5, Enzyme: 10.1 Ug^−1^	[56]
Sisal Waste	EAE	50	1200	15	Enzyme: 88 Ug^−1^, pH:5	[57]
Tobacco waste	MAE	-	4	20	550 W, pH: 1.8	[58]
Cocoa Pod Husk waste	SWE	121	30	27.5	103.4 bar	[59]
Custard apple peel waste	UAE	63	18	21	pH: 3	[60]
Mango peel waste	UAE	85	10	7.6	497.4 W/cm^2^, pH: 2	[61]
Sisal Waste	UAE	-	60	15	450W, pH: 4	[57]
Passion fruit rind	UAE	-	10	20	135W	[62]
Jackfruit peel waste	UAE-MAE	86	29	48	-	[63]

HAE: Hydrothermal-assisted extraction; UAE: Ultrasound-assisted extraction; HC: Hydrodynamic cavitation; MAE: Microwave-assisted extraction; SWE: Subcritical water extraction; EAE: Enzyme-assisted extraction.

**Table 2:** Different types of pectin-based materials used in food packaging applications.

Type	Polymer Matrix	Additive	Application	Ref.
Film	LMP-bitter vetch protein	Transglutaminase	Drug delivery system	[72]
Film	LMP	Ascorbic acid	AO system	[73]
Film	HMP	Clove EO	AM system	[74]
Film	HMP	Marjoram EO	AO system	[75]
Film	HMP-Gluconaman	Tea extract	AO/AM system	[76]
Film	Pectin-Pullulan	AgNPs	AM system	[77]
Film	HM-Apple pectin	Chia seed hydrocolloid	AO system	[10]
Film	Chitosan-Starch-Pectin	Mint and rosemary oils	AO/AM system	[78]
		Nisin		
Film	Fish gelatine-HMP	Hydroxytyrosol, dihydroxyphenylglycol	Preservation of beef meat	[79]
Film	HMP	Red cabbage extract	pH indicator	[80]
Film	Chitosan-HMP	Anthocyanin	pH indicator	[81]
Nanocomposite	Pectin	AgNPs, laponite	Coating polypropylene to improve barrier/AM properties	[82]
Nanocomposite	Pectin	Ag/AgCl-ZnONPs	AM system	[83]
Nanofiber	HMP	AgNPs	Reinforcement, AM	[84]
Nanofiber	LMP Polyethylene oxide	-	Reinforcement	[85]
Aerogel	Amidated pectin	TiO_2,_ NPs	AM under dark and UV illumination conditions	[86]
Hydrogel	LMP-Chitosan	Garlic and holy basil EOs	Incorporate to cellulose bag to improve AM properties	[87]
Oleogel	HMP	Camelia oil Tp-Palmitate	Drug delivery system	[88]
Emulsion	HMP	Clove EO	Bream fillets coating	[89]
Microemulsion	Chitosan-HMP	Cinnamaldehyde	AM system	[90]
Nanoemulsion	Food-grade pectin	Curcumin and garlic EOs	Coating chicken fillets	[91]
Nanoemulsion	HMP	Oregano, thyme, lemongrass, mandarin EOs	AM system	[92]
Nanoemulsion	HMP	Lemongrass EO	Addition in Cassava starch film to improve biodegradation properties	[93]
Multilayer emulsion	HMP-Chitosan	Astaxanthin	Release of hydrophobic carotenoids	[94]

EO: essential oil; AM: antimicrobial; AO: antioxidant.
